# Neonatal bacterial meningitis versus ventriculitis: a cohort-based overview of clinical characteristics, microbiology and imaging

**DOI:** 10.1007/s00431-020-03723-3

**Published:** 2020-07-03

**Authors:** Thomas Peros, Joost van Schuppen, Anneloes Bohte, Caspar Hodiamont, Eleonora Aronica, Timo de Haan

**Affiliations:** 1 Department of Pediatric Intensive Care, Amsterdam University Medical Centre, Amsterdam, Netherlands; 2 Department of Radiology and Nuclear Medicine, Amsterdam University Medical Centre, Amsterdam, Netherlands; 3 Department of Radiology and Nuclear Medicine, University Medical Centre Utrecht, Utrecht, Netherlands; 4 Department of Microbiology, Amsterdam University Medical Centre, Amsterdam, Netherlands; 5 Department of (Neuro) Pathology, Amsterdam University Medical Centre, Amsterdam, Netherlands; 6 Department of Neonatal Intensive Care, Amsterdam University Medical Centre, Amsterdam, Netherlands

**Keywords:** Ventriculitis, CNS infections, Meningitis, Neonate, Cranial ultrasound

## Abstract

Central nervous system (CNS) infections are potentially life threatening in neonates and can lead to the ill-defined diagnosis of ventriculitis. With this study we aimed to explore and describe ventriculitis regarding clinical, microbiological and ultrasonographic characteristics. We performed a retrospective cohort study including all neonates with a culture-proven CNS infection admitted to our tertiary NICU over a 12-year period (2004–2016). For each case clinical data was gathered, and three timed cranial ultrasounds were anonymized and retrospectively reviewed and assessed for signs of ventriculitis. Forty-five patients were included with 9 (20%) diagnosed with ventriculitis. Mortality in both ventriculitis and non-ventriculitis cases was one-third. Patients with pre-existing conditions as post-haemorrhagic hydrocephalus are at risk of developing ventriculitis. Most common pathogens were gram negative bacteria (68.9%). Ultrasonographic signs of ventriculitis developed over time, and interrater agreement was substantial.

*Conclusion*: Neonatal ventriculitis is a serious entity in the continuum of meningitis. Early and correct diagnoses of ventriculitis are both important because of possible persisting or newly developing hydrocephalus or seizures. Sequential imaging should be performed.

**Table:** 

**What is Known:** *• CNS infections in neonates lead to high mortality and morbidity.* *• Ventriculitis is a severe complication of meningitis.*	
**What is New:** *• High morbidity; the majority of ventriculitis patients have pre-existing PHVD and develop seizures and hydrocephalus.* *• Interrater agreement is good; bedside CUS is a useful tool for reaching a sustainable diagnosis of ventriculitis.*

**Electronic supplementary material:**

The online version of this article (10.1007/s00431-020-03723-3) contains supplementary material, which is available to authorized users.

## Introduction

Sepsis and CNS infections are severe complications in the neonatal and infant period. Despite ongoing advances in neonatal care, the prevalence of neurological sequelae following neonatal CNS infections remains high [1–4]. According to the Annual Report of the Netherlands Reference Laboratory for Bacterial Meningitis of the Amsterdam University Medical Centre and the National Institute of Public Health and the Environment (RIVM), the 2015 and 2016 incidence of neonatal CNS infection in the Netherlands has been 0.3 cases per 1000 live births [5]. This is comparable with the UK and Ireland where the incidence is estimated at 0.38 per 1000 live births [2]. The most common causative organisms of neonatal CNS infections are *group B streptococci* and *Escherichia coli* [2, 3, 6].

One of the severe short-term complications of CNS infection is inflammation of the ventricular system or ventriculitis [1, 7]. Little is known about the pathophysiology and incidence of ventriculitis, and there is no clear definition for diagnosis. An early diagnosis however appears relevant, since neurological complications can occur [8, 9].

A tool for early diagnosis could be the use of bedside cranial ultrasound (CUS). Although several characteristic findings on CUS suggestive of ventriculitis have been described, there are no definitive radiological criteria defined in the literature leading to possible under- or overdiagnosis.

Previously described CUS findings in ventriculitis are irregular thickening and increased echogenicity of the ventricular lining and intraventricular debris or stranding. This organization of exudate in the ventricles may lead to the formation of cysts, compartmentalisation or obstructive hydrocephalus [10–12]. However, the presence of intraventricular blood, common in premature neonates, might interfere with diagnosing ventriculitis due to overlap in ultrasonographic findings.

With this study we aim to explore and describe the entity of ventriculitis. We aim for comparison between CNS infections with and without ventriculitis regarding clinical, microbiology and ultrasonographic characteristics. Furthermore, we aim to retrospectively review all available ultrasound imaging results performed in our cohort to describe typical findings and establish the interrater agreement diagnosing ventriculitis.

## Methods

### Case identification and clinical characteristics

This study was setup as a retrospective cohort study. After consulting our local microbiology department database, we identified and included all cases with a (CSF) culture-proven CNS bacterial infection admitted to our tertiary NICU between 2004 and 2016. During that period it was standard care for all NICU patients with a clinical suspicion of sepsis to undergo a lumbar puncture before starting antimicrobial therapy. Cases with congenital abnormalities of the brain as well as cases with contaminated (false positive) cultures were excluded.

Patients were divided into two groups, CNS infections with signs of ventriculitis (from here on; ventriculitis) and CNS infections without ventriculitis (from here on; meningitis). The diagnosis ventriculitis was based on the following CUS findings: abnormal ventricular lining (echogenicity or thickening) and/or abnormal CSF (debris or stranding visible).

For all included cases, data regarding birth conditions, comorbidities, symptoms and timing of diagnosis, illness severity, required support, complications and laboratory results were collected from the hospital information system and subsequently analysed.

#### Microbiology

Cultures were taken prior to start of any antimicrobial therapy. CSF samples were cultured both in broth and on solid culture media (on Columbia sheep blood agar and chocolate agar incubated at 37 °C, in an incubator enriched with 5% CO2, and on Columbia sheep blood agar at 37 °C in anaerobic conditions) for a total of 7 days. Identification of cultured microorganisms was performed using biochemical tests prior to the introduction of MALDI-TOF MS in 2010 (Biotyper, Bruker Daltonics, Bremen, Germany).

#### Cranial ultrasounds and interrater agreement

CUS were performed using a Siemens Sonoline Elegra Ultrasound (Siemens Healthineers, Erlangen, Germany) or a Philips iU22 ultrasound system (Phillips Healthcare, Best, The Netherlands). A dedicated 8–5 MHz broadband curved array probe was used. CUS were requested at the time because of scheduled clinical follow-up protocol or were performed on clinical indication (i.e. any clinical increase in illness severity; increase in head circumference or clinical seizures). Standard coronal and sagittal views, including transcranial Doppler ultrasound, were made and stored on the PACS system; the report and diagnosis were stored in the hospital information system.

We selected 3 consecutive CUS of acceptable imaging quality for each case: CUS1, prior to positive CSF culture date (baseline); CUS2, closest in time point to the first positive CSF culture date (at diagnosis); and CUS3, 1 week after positive CSF culture date (post diagnosis).

The images were collected, anonymized and offered for secondary review to AB, fellow paediatric radiology. This reviewer was not aware of the clinical information and the goals of this study; the diagnosis ventriculitis was not mentioned prior to review of all the images. The reviewer was asked to describe the CUS, using a standardized ultrasound checklist (Appendix 1); this checklist included general features of CUS and possible signs of ventriculitis as derived from the available literature. To assess the interrater agreement, the blinded secondary review was then compared with the initial review by the performing or supervising paediatric radiologist.

#### Statistics

Statistical analyses were performed with SPSS 26.0 statistical software package (SPSS Inc., Chicago, IL). For normally distributed data, mean and standard deviations were calculated and reported and for skewed data the median and range.

For comparison between proportions in the meningitis and ventriculitis groups in Table 1, two-sided Fisher Exact test was used. To compare means in the laboratory results, an independent samples *t*-test was used. To assess the differences between ultrasound findings of the specified time points (comparing CUS1 with CUS2 and CUS2 with CUS3), the Pearson Chi-square test was used.

**Table 1:** Patient characteristics, comorbidities, symptoms and illness severity

		Meningitis (*n* = 36)	Ventriculitis (*n* = 9)	All (*n* = 45)	*p*
Patient characteristics					
Gestational age	1	28.0 (24.7–40.7)	27.4 (25.4–30.0)	28.0 (24.7–40.7)	**–**
Birth weight (grams)	1	1110 (585–4690)	895 (725–1570)	1090 (585–4690)	–
Male		21 (58.3%)	4 (44.4%)	25 (55.6%)	–
Twin		5 (13.8%)	1 (11.1%)	6 (13.3%)	–
APGAR < 5 at 5 min		4 (11.1%)	1 (11.1%)	5 (11.1%)	–
Birth defects		3 (8.3%)	0 (0%)	3 (6.7%)	–
Antibiotics after birth in days	1	2 (0–14)	3 (1–7)	2 (0–14)	–
No antenatal steroids		10 (27.7%)	2 (22.2%)	12 (26.7%)	–
Diagnosis and pre-existing comorbidities					
Weight at diagnosis	1	1090 (650–4380)	1010 (870–2360)	1080 (650–4380)	–
Age (days) at diagnosis	4	11.8 (± 9.4)	34.7 (± 32.1)	16.4 (± 18.5)	–
CVL > 1 day at diagnosis	2	23 (63.8%)	2 (22.2%)	25 (55.6%)	0.030
PDA	2	8 (22.2%)	6 (66.6%)	14 (31.1%)	0.002
PDA requiring treatment	2	5 (13.8%)	4 (44.4%)	9 (20%)	–
Surgical comorbidity (including NEC)		10 (27.7%)	3 (33.3%)	13 (28.9%)	–
Prolonged respiratory support		6 (16.6%)	3 (33.3%)	9 (20%)	–
Fully TPN dependant		7 (19.4%)	1 (11.1%)	8 (17.8%)	–
PHVD requiring drainage	2	2 (5.55%)	4 (44.4%)	6 (13.3%)	0.010
Symptoms at diagnosis					
Apneas/bradycardia		21 (58.3%)	2 (22.2%)	23 (51.1%)	–
Neurological symptoms		2 (5.5%)	1 (11.1%)	3 (6.7%)	–
GI symptoms		6 (16.6%)	1 (11.1%)	7 (15.6%)	–
Circulatory failure		16 (44.4%)	2 (22.2%)	18 (40%)	–
Respiratory failure		20 (55.5%)	4 (44.4%)	24 (53.3%)	–
Skin lesions		3 (8.33%)	0 (0%)	3 (6.7%)	–
Fever		1 (2.7%)	1 (11.1%)	2 (4.4%)	–
Support required					
Non-invasive respiratory support	3	7 (19.4%)	2 (22.2%)	9 (20%)	–
Invasive ventilation	3	25 (69.4%)	5 (55.5%)	30 (66.7%)	–
Fluid resuscitation	3	8 (22.2%)	3 (33.3%)	11 (24.4%)	–
Inotropic support	3	16 (44.4%)	2 (22.2%)	18 (40%)	–
Post diagnosis complications					
Hydrocephalus (requiring treatment)	2	1 (2.77%)	5 (55.5%)	6 (13.3%)	0.001
Seizures	2	6 (16/6%)	4 (44.4%)	10 (22.2%)	0.017
Cerebral abscess	2	1 (2.77%)	2 (22.2%)	3 (6.7%)	–
Death		12 (33.3%)	3 (33.3%)	15 (33.3%)	–
Laboratory results					
CRP (mg/l)	1	47.3 (0–265.1)	69.2 (0–260.8)	49.3 (0–265.1)	–
Platelets (× 10e9/L)	1	130 (0–534)	125 (30–403)	128 (0–534)	–
WBC (× 10e9/L)	1	13 (1.5–42.1)	11.1 (5.3–29.4)	13 (1.5–42.1)	–
CSF WBC (× 10e6/L)	1	102 (1–51.200)	1376 (141–43.410)	144 (1–51.200)	–
CSF protein (g/L)	2, 4	1.98 (± 1.33)	3.58 (± 2.72)	2.23 (± 1.67)	0.029
CSF glucose (mmol/l)	2, 4	3.68 (± 2.29)	1.13 (± 1.08)	3.43 (± 2.32)	0.009
CSF WBC polymorphonuclear %	4	47.4% (± 20.8%)	68.7% (± 4.3%)	51.0% (± 20.8%)	–
CSF WBC mononuclear %	4	52.6% (± 20.9%)	31.3% (± 4.3%	49.0% (± 20.8%)	–

1, Values are medians and range. 2, Significant difference between meningitis and ventriculitis. 3, Support required any time after onset of illness. 4, Values are mean and standard deviation

To evaluate the measure of agreement between the reviewing radiologists reports with the primary diagnosis (dichotomic diagnosis ventriculitis: Yes/No) Cohen’s kappa (*k*) was calculated. A *k* < 0 reflects ‘poor’, 0 to 0.20 ‘slight’, 0.21 to 0.4 ‘fair’, 0.41 to 0.60 ‘moderate’, 0.61 to 0.8 ‘substantial’ and above 0.81 ‘almost perfect agreement’. To further assess interrater agreement, we determined the intra-class correlation (ICC). The ICC assesses rating reliability by comparing the variability of different ratings of the same subject to the total variation across all ratings and all subjects. In our cohort the raters were fixed and subject a random sample. We therefore assessed the ICC with a two-way mixed model for absolute agreement.

## Results

### Cases and clinical characteristics

We included 45 patients with a culture-proven CNS infection. Nine patients were diagnosed as having ventriculitis; 36 patients were diagnosed with meningitis. A total of 79 cases were excluded because CSF cultures were considered contaminated (false positive) samples after review of the case notes. Those patients were not considered and treated as CNS infections at the time. The patient characteristics, timing of and symptoms at diagnosis, comorbidities, complications, laboratory results and illness severity of both meningitis and ventriculitis groups are as shown in Table 1.

There were no significant differences in patient characteristics. The age in days at diagnosis shows a non-significantly later onset of the ventriculitis cases (34.7 days versus 11.8 days, *p* = 0.061). Larger proportions of patients in the meningitis group had a CVL (> 1 day) in situ at diagnosis. In the ventriculitis group, significantly more patients had a PDA (6/9 versus 8/36); however, further narrowing these patients to PDA requiring intervention (medication or clip) the difference lost statistical significance (*p* 0.06). No differences were found regarding presenting symptoms. In the ventriculitis cases, 4/9 had pre-existing hydrocephalus due to earlier haemorrhage (PHVD) with 2 out of these 4 having CSF drainage devices in situ (Omaya drain). Eventually 5/9 patients developed significant hydrocephalus. Seizures were more frequent in ventriculitis patients. Mortality was similar for patients with or without ventriculitis (33.3%). Laboratory results showed no difference in CRP, full blood count results or cell count in CSF. CSF protein was higher, and CSF glucose was lower in ventriculitis patients.

### Microbiology

The causative organisms found in both the ventriculitis and CNS infection group are shown in Table 2 and were similar in both groups. The most common pathogens we found were gram negative rods (68.9%), most importantly *Escherichia coli* (22.2%), *Klebsiella pneumoniae* (17.8%) and *Enterobacter cloacae* (13.3%). Gram positive bacteria accounted for about one-third of the pathogens with coagulase-negative staphylococci being the fourth most common (11.1%). Group B streptococci were found in 6.7% of our cohort. Mortality in both the gram positive (28.6%) and gram negative (35.5%) group was comparable. There were no significant differences between the found pathogens (or grouped as gram+ versus gram-) and the frequency of the required support or the development of post diagnosis complications as mentioned in Table 1.

**Table 2:** Pathogens

	Meningitis		Ventriculitis		All	
	(*n* = 36)		(*n* = 9)		(*n* = 45)	
	*n*	%	*n*	%	*n*	%
Coagulase negative staphylococci	3	8.3%	2	22.2%	5	11.1%
*Enterococcus faecalis*	4	11.1%	0	0.0%	4	8.9%
Group B streptococcus	3	8.3%	0	0.0%	3	6.7%
*Staphylococcus aureus*	1	2.8%	1	11.1%	2	4.4%
*Gram Positive*	*11*	30.6%	*3*	33.3%	*14*	*31.1%*
*Escherichia coli*	7	19.4%	3	33.3%	10	22.2%
*Klebsiella pneumoniae*	7	19.4%	1	11.1%	8	17.8%
*Enterobacter cloacae*	4	11.1%	2	22.2%	6	13.3%
*Klebsiella oxytoca*	4	11.1%	0	0.0%	4	8.9%
*Enterobacter aerogenes*	2	5.6%	0	0.0%	2	4.4%
*Serratia marcescens*	1	2.8%	0	0.0%	1	2.2%
*Gram Negative*	*25*	69.4%	*6*	66.7%	*31*	*68.9%*

No significant difference between meningitis and ventriculitis groups

### Cranial ultrasounds and interrater agreement

**Fig. 1:**
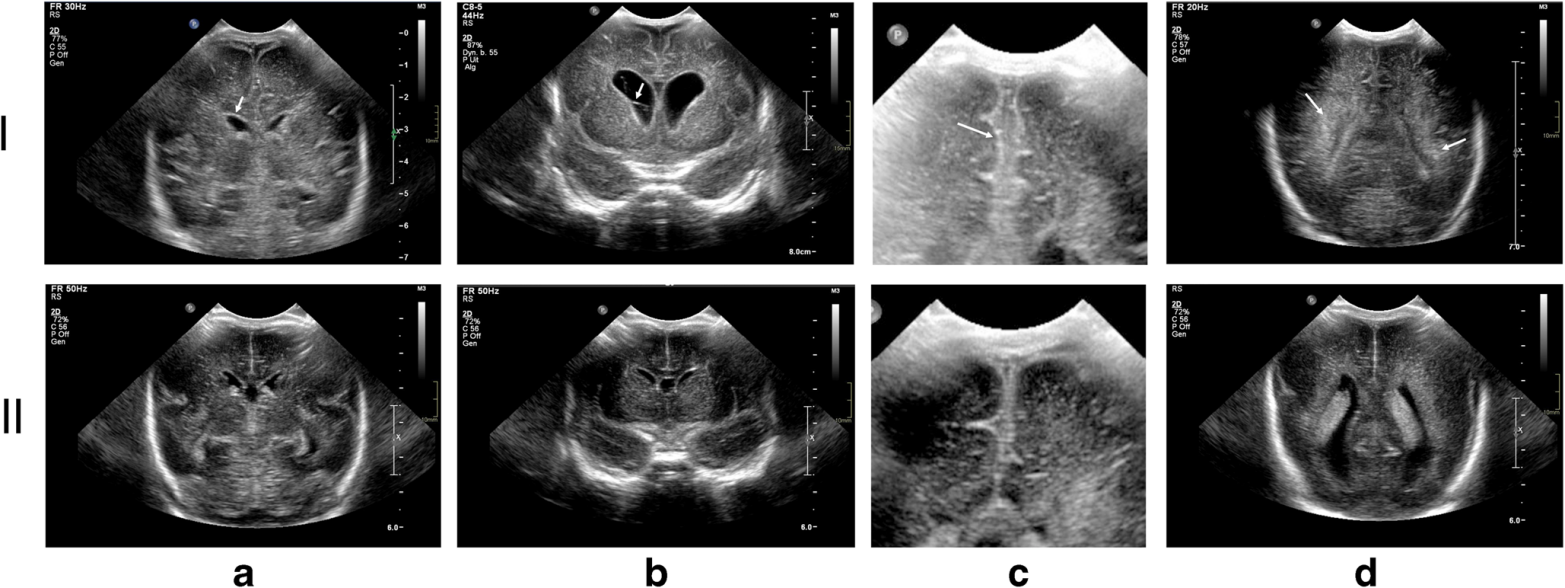
Ultrasound imaging from our cohort. (I) Top row shows images from ventriculitis patients. (II) Bottom row shows contrasting images from meningitis patients. IA: Slightly thickened ultrasound rich ventricle walls. IB: Coronal image of bilateral enlarged ventricles, with stranding in the right ventricle. IC: Coronal image showing hyperechoic lining of the gyri and sulci, hyperechoic peripheral CSF. ID: Hyperechoic appearance of white matter. IIA: Normal periventricular lining. IIB: Normal non-reflecting CSF. No hydrocephalus. IIC: Virtual normal lining. IID: Normal white matter surrounding the ventricles

The ultrasonographic characteristics as described in the secondary review of the CUS are shown in Table 3. In total 111 of the expected 135 CUS (*n* = 45 × 3 CUS) were available for secondary review. In 6 out of 45 cases, a baseline CUS1 was not available because it was not required due to protocol (GA > 32 weeks) or the patient was diagnosed with a CNS infection on day one of life. In 4 cases CUS2 was not available, and in 14 cases CUS3 was not available (9 patients died and 5 were transferred to a referral hospital). Figure 1 shows exemplary CUS findings in our ventriculitis cases contrasted to meningitis cases from our cohort.

**Table 3:** Ultrasound findings and time course

	CUS1 (*n* = 39)	CUS2 (*n* = 41)	CUS3 (*n* = 31)	Sign (1–2)	Sign (2–3)
Abnormal CSF (ventricular) ±	5% (2)	15% (6)	26% (8)	0.030*	0.000*
Abnormal CSF (peripheral) ±	5% (2)	10% (4)	13% (4)	0.000*	0.042*
Abnormal ventricular lining ±	5% (2)	10% (4)	16% (5)	0.504	0.028*
Hydrocephalus	8% (3)	15 (6)	26% (8)	0.002*	0.063
IVH	85% (33)	73% (30)	77% (24)	0.120	0.218
Flaring	15% (6)	15% (6)	0% (0)	0.331	0.315
Parenchymal focal/diffuse	0% (0)	7% (3)	10% (3)	0.057	0.562
Echogenic lining of gyri	10% (4)	7% (3)	3% (1)	0.220	0.234
Abnormal shape of gyration	15% (6)	17% (7)	13% (4)	0.423	0.127
Other: Ischaemia	0% (0)	7% (3)	6% (2)	0.209	0.004*
Other: Infarction	3% (1)	7% (3)	0% (0)	0.489	0.000*
Other: Increased RI	8% (3)	17 (7)	26% (8)	0.257	0.168
Other: Oedema	0% (0)	0% (0)	3% (1)	0.197	0.245
Other: Abscess	0% (0)	2% (1)	3% (1)	0.288	0.149

**±**Diagnostic criteria for ventriculitis

*CUS1*, baseline; *CUS2* at diagnosis, *CUS3* 1 week post diagnosis

Sign (1–2): *p* value of Pearson Chi-square comparing difference between CUS1 and CUS2 finding

Sign (2–3): *p* value of Pearson Chi-square comparing difference between CUS2 and CUS3 finding

*Indication of significance

All nine ventriculitis cases were confirmed by the secondary reviewer. The Cohen’s kappa for agreement between initial and secondary (anonymised) review was 0.70 (SE 0.137), *p* < 0.001. The ICC taking into account group and subject variability was 0.50 (CI 0.09–0.71), *p* 0.01.

To illustrate the different facets of ventriculitis in a single patient, we have added supplementary material containing CUS imaging, MRI and pathology findings from a premature patient with ventriculitis.

## Discussion

### Cases and clinical characteristics

Our study presents a detailed cohort of NICU-admitted neonates with culture-proven CNS infections, exploring and comparing ventriculitis and meningitis with regard to clinical, microbiology and ultrasonographic characteristics. We found that ventriculitis is a common complication and appears to lie in the continuum of bacterial CNS infections in neonates.

A significant part of our cohort with CNS infections developed ventriculitis (20%). This is comparable with the available literature. Two Taiwanese retrospective cohort studies, published in 2014 and 2018, found ventriculitis to develop in 15.3–20.8% of neonatal CNS infection patients [1, 7]. Ouchenir et al. demonstrated ventriculitis in 6.3% of all patients in a Canadian neonatal CNS infection cohort. However, a critical review of the imaging results in this cohort (a combination of CUS, CT scan, MRI) raised the suspicion for ventriculitis in 13% of patients, more comparable with the results in our study [3].

Both meningitis and ventriculitis proved to be serious conditions with a high morbidity and mortality. Mortality in our cohort was one-third in both ventriculitis and meningitis. Kumar et al. found a mortality of 22.2% in a ventriculitis cohort at long-term follow-up (10/45 cases) [8]. Two British and French studies found the mortality of neonatal CNS infection to be 8–13%. Both studies found the mortality to be significantly higher in preterm and very preterm infants (up to 27%), which is comparable with the mortality in our cohort with a mean GA of around 28 weeks [2, 6].

We found significantly more ventriculitis patients to have a PDA; however, further narrowing to hemodynamically significant PDA requiring treatment statistical significance was lost. A possible explanation is the relationship between PDA and IVH (subsequently PHVD being more common in ventriculitis) with ductal steal leading to less cerebral flow increasing the risk of IVH [13, 14].

The increased presence of CVL in meningitis patients is possibly correlated to the (non-significantly) earlier diagnosis of the CNS infection in this group. In our experience the majority of patients in initial NICU management (first 2–3 weeks after birth) have a CVL in situ for TPN or medication. Patients developing ventriculitis show a trend to be weeks older and possibly had less need for a CVL at the time of onset.

Complications in the ventriculitis group were significantly higher. We found that more patients at some point developed seizures and the majority of cases developed a significant hydrocephalus needing treatment (versus only 2.6% in the meningitis group). Our findings were comparable with that of an Indian cohort describing hydrocephalus in 50% of ventriculitis cases in 2015 [8]. However, 4 out of 5 ventriculitis patients with hydrocephalus in our cohort had a pre-existing PHVD requiring treatment with either LP or a drainage device.

It appears that the presence of IVH with required repeated drainage of CSF is possibly associated with the development of ventriculitis. We cannot however with our limited data substantiate this finding.

The repeated therapeutic CSF drainage procedures in IVH patients could be an entry point for pathogens leading to iatrogenic CNS infection advancing to include the ventricles. Based on our findings, patients with PHVD are at risk for development of ventriculitis, and in reverse patients with known ventriculitis should be closely monitored for developing hydrocephalus.

CSF composition comparison showed (non-significantly) higher leukocyte count in ventriculitis cases. We found a significantly higher CSF protein and lower CSF glucose value in ventriculitis cases. Higher WBC and protein in CSF are associated with bacterial CNS infections [15]. Possibly the ongoing bacterial infection in ventriculitis is eventually leading to the higher values we found. Ideally we would prefer to examine the possible differences in the blood/CSF glucose ratio, but these data were not available.

### Microbiology

The pathogens cultured in our cohort are common causative pathogens in the NICU environment. We found *E. coli*, *Klebsiella pneumoniae* and *Enterobacter cloacae* to be the three most commonly encountered pathogens our cohort. Other studies however have shown *GBS* to be the most common pathogen, followed by *E. coli* and other bacteria [2, 3, 6]. Oikike et al. demonstrated *E. coli* to be equally prevalent to *GBS* in hospitalized preterm infants during CNS infection [2]. Our cohort consisted of early preterm infants, and these are especially susceptible to severe acquired gram negative nosocomial pathogens like *Klebsiella* and *Enterobacter* species [16, 17].

We could not find any significant differences between pathogens (individual or grouped as gram + and gram-) and mortality, disease severity or post diagnosis complications. Regarding pathogens and mortality, there have been some contrasting findings in previous literature with a study in 2004 finding higher mortality in gram negative CNS infections [18] and a study published in 2006 finding no significant differences between gram negative and positive infections CNS infections [19].

### Cranial ultrasounds and interrater agreement

In our study we used the presence of increased echogenic lining of the ventricle, ventricular debris, visible strands and ventricular dilatation as findings consistent with a diagnosis of ventriculitis. This corresponds with the available literature on ultrasound findings in ventriculitis patients [12, 20]. We established that neonatal CNS infections gradually develop to ventriculitis over time by observing a significant increase of the ultrasonographic ventriculitis characteristics in each consecutive CUS.

The time course also demonstrated that areas suspect for ischemia or infarction were more often seen on the last (CUS3) ultrasound. Patients with CNS infections should therefore be monitored for cerebral insult. At the time the patients of our cohort were admitted, MRI investigations were not performed in meningitis/ventriculitis cases as a standard of care. In current times, if the clinical condition permits, we strongly recommend more detailed additional imaging.

The interrater agreement between paediatric radiologists in the diagnosis of ventriculitis proved high. Our Cohen’s kappa value points to a substantial agreement according to Landis and Koch [21]. Correcting for group and subject variability (assessing the ICC) however decreased the measure of agreement statistically. The CI of the ICC was wide due to the small number of cases, but the upper margin of the confidence interval (0.71) was comparable with the value of the Cohen’s kappa value (0.70). To reliably assess the interrater agreement of the use of ultrasound in this disease, a larger group of cases would be needed. As neonatal CNS infections are infrequent but serious (and larger case numbers may be difficult to attain), we state that bedside ultrasound is a reliable tool for the diagnosis for ventriculitis.

### Limitations

Although our study was performed in a unique cohort, there are several limitations to this study and its results. Due to the limited numbers, we were unable to determine reliable corrected risk factors regarding comorbidities.

As mentioned, we would preferably have had access to (sequential) cerebral MRI as a golden standard for ventriculitis at the moment of the CNS infection in our cohort. Beyond more detailed and valuable information, it would have given us the possibility of calculating sensitivity and specificity of CUS and ventriculitis. However, the logistic challenges due to the critical condition of a significant number of the patients made MRI scanning less accessible.

The retrospective nature interfered with the quality of the available imaging as it was not always consistent with the high imaging standards we adhere today. Additionally, reassessing static ultrasound images is less optimal to dynamic assessment. Finally, similarities in findings in ventriculitis and IVH can make the correct diagnosis more challenging possibly leading to under (or over-) diagnosis of ventriculitis.

Despite these limitations, we strongly believe our results are of importance for clinicians, attending neonatologists and paediatric radiologists. Future prospective studies should focus on high quality sequential cerebral ultrasound and MR imaging during neonatal CNS infection. Imaging results should be combined with detailed and standardized long-term neurodevelopmental outcome assessments to identify important risk factors for outcome or disease progression.

## Conclusions

Neonatal ventriculitis is a serious entity in the continuum of meningitis. Patients with pre-existing conditions like PHVD are at risk of developing ventriculitis. Early and correct diagnoses of ventriculitis are both important because of possible persisting or newly developing hydrocephalus or seizures. Due to the progressive nature of meningitis to ventriculitis, sequential imaging of neonates with CNS infections should be performed.

Bedside cerebral ultrasound proved to be a reliable tool for this sequential imaging leading to a sustainable radiological diagnosis of ventriculitis. Criteria to diagnose ventriculitis should include increased echogenic lining of the ventricle, ventricular debris, visible strands and ventricular dilatation.

## Electronic supplementary material

ESM 1 (DOCX 264 kb).

## Data Availability

We have conducted a single centre study and therefore no data was shared with other centres. The data are not publicly available due to them containing information that could compromise research participant privacy/consent. The data are available on (reasonable) request from the corresponding author (TP).

## Abbreviations

BW: Birth weight
CUS: Cranial ultrasound scan
CVL: Central venous line
GA: Gestational age
GBS: Group B streptococci
IVH: Intraventricular haemorrhage
NEC: Necrotizing enterocolitis
PDA: Patent ductus arteriosus
PHVD: Post haemorrhagic ventricular dilatation
RI: Intracranial resistive index
TPN: Total parenteral nutrition

## References

[1] Chu SM, Hsu JF, Lee CW, Lien R, Huang HR, Chiang MC, Fu RH, Tsai MH (2014). Neurological complications after neonatal bacteremia: the clinical characteristics, risk factors, and outcomes. PLoS One.

[2] Okike IO, Johnson AP, Henderson KL, Blackburn RM, Muller-Pebody B, Ladhani SN, Anthony M, Ninis N, Heath PT, neoMen Study G (2014). Incidence, etiology, and outcome of bacterial meningitis in infants aged <90 days in the United Kingdom and Republic of Ireland: prospective, enhanced, national population-based surveillance. Clin Infect Dis.

[3] Ouchenir L, Renaud C, Khan S, Bitnun A, Boisvert AA, McDonald J, Bowes J, Brophy J, Barton M, Ting J, Roberts A, Hawkes M, Robinson JL (2017) The epidemiology, management, and outcomes of bacterial meningitis in infants. Pediatrics 140(1). 10.1542/peds.2017-0476 10.1542/peds.2017-0476 28600447

[4] Bedford H, de Louvois J, Halket S, Peckham C, Hurley R, Harvey D (2001). Meningitis in infancy in England and Wales: follow up at age 5 years. BMJ.

[5] Van der Ende Aea Bacterial meningitis in the Netherlands; annual report 2015+2016. Netherlands Reference Laboratory for Bacterial Meningitis (AMC/RIVM),

[6] Gaschignard J, Levy C, Romain O, Cohen R, Bingen E, Aujard Y, Boileau P (2011). Neonatal bacterial meningitis: 444 cases in 7 years. Pediatr Infect Dis J.

[7] Hsu MH, Hsu JF, Kuo HC, Lai MY, Chiang MC, Lin YJ, Huang HR, Chu SM, Tsai MH (2018). Neurological complications in young infants with acute bacterial meningitis. Front Neurol.

[8] Kumar R, Singhi P, Dekate P, Singh M, Singhi S (2015). Meningitis related ventriculitis--experience from a tertiary care centre in northern India. Indian J Pediatr.

[9] Miyairi I, Causey KT, DeVincenzo JP, Buckingham SC (2006). Group B streptococcal ventriculitis: a report of three cases and literature review. Pediatr Neurol.

[10] Gupta N, Grover H, Bansal I, Hooda K, Sapire JM, Anand R, Kumar Y (2017). Neonatal cranial sonography: ultrasound findings in neonatal meningitis-a pictorial review. Quant Imaging Med Surg.

[11] Nickerson JP, Richner B, Santy K, Lequin MH, Poretti A, Filippi CG, Huisman TA (2012). Neuroimaging of pediatric intracranial infection--part 1: techniques and bacterial infections. J Neuroimaging.

[12] Yikilmaz A, Taylor GA (2008). Sonographic findings in bacterial meningitis in neonates and young infants. Pediatr Radiol.

[13] Perlman JM, Hill A, Volpe JJ (1981). The effect of patent ductus arteriosus on flow velocity in the anterior cerebral arteries: ductal steal in the premature newborn infant. J Pediatr.

[14] Meek JH, Tyszczuk L, Elwell CE, Wyatt JS (1999). Low cerebral blood flow is a risk factor for severe intraventricular haemorrhage. Arch Dis Child Fetal Neonatal Ed.

[15] de Blauw D, Bruning A, Vijn LJ, Wildenbeest JG, Wolthers KC, Biezeveld MH, van Wermeskerken AM, Nauta F, Pajkrt D (2019). Blood and cerebrospinal fluid characteristics in neonates with a suspected central nervous system infection. Medicine (Baltimore).

[16] Pokhrel B, Koirala T, Shah G, Joshi S, Baral P (2018). Bacteriological profile and antibiotic susceptibility of neonatal sepsis in neonatal intensive care unit of a tertiary hospital in Nepal. BMC Pediatr.

[17] Softic I, Tahirovic H, Di Ciommo V, Auriti C (2017). Bacterial sepsis in neonates: single Centre study in a neonatal intensive care unit in Bosnia and Herzegovina. Acta Med Acad.

[18] Stoll BJ, Hansen N, Fanaroff AA, Wright LL, Carlo WA, Ehrenkranz RA, Lemons JA, Donovan EF, Stark AR, Tyson JE, Oh W, Bauer CR, Korones SB, Shankaran S, Laptook AR, Stevenson DK, Papile LA, Poole WK (2004). To tap or not to tap: high likelihood of meningitis without sepsis among very low birth weight infants. Pediatrics.

[19] Smith PB, Cotten CM, Garges HP, Tiffany KF, Lenfestey RW, Moody MA, Li JS, Benjamin DK (2006). A comparison of neonatal gram-negative rod and gram-positive cocci meningitis. J Perinatol.

[20] Tatsuno M, Hasegawa M, Okuyama K (1993). Ventriculitis in infants: diagnosis by color Doppler flow imaging. Pediatr Neurol.

[21] Landis JR, Koch GG (1977). The measurement of observer agreement for categorical data. Biometrics.

