# Supplementary material for: Neonatal bacterial meningitis versus ventriculitis: a cohort-based overview of clinical characteristics, microbiology and imaging
Source: Eur J Pediatr. 2020 Jul 3;179(12):1969–77. doi: 10.1007/s00431-020-03723-3 (PMC7666663; doi:10.1007/s00431-020-03723-3)
Supplement: Supplementary file 1 — (DOCX 264 kb). [file 431_2020_3723_MOESM1_ESM.docx]

**Case information**

This case concerned a female preterm born neonate of 28 weeks gestational age. No perinatal asphyxia was described. There was an uncomplicated clinical course up until the postnatal age of 7 days when a clinical sepsis with CNS infection- ventriculitis occurred caused by Klebsiella Oxytoca. The clinical course was also complicated by a bilateral grade 3 IVH and a large cerebellar haemorrhage with ensuing hydrocephalus, requiring drainage. The patient died 20 days after birth.

**Imaging**


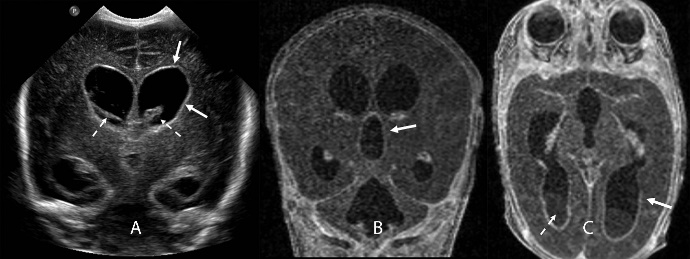


**Figure 2**

1. Coronal CUS. Clearly visible hydrocephalus of all ventricles. Hyperechogenic thickening of the ventricles is seen in both lateral ventricles and in the third ventricles (white arrow). Furthermore, intraventricular debris and stranding is appreciated. (dashed arrows)
2. MRI imaging in same patient; Coronal post gadolinium T1, comparable with position of ultrasound. Hydrocephalus is seen. Additionally, the ependyma surrounding the third ventricle shows attenuation post contrast. (arrow)
3. MRI imaging in same patient; Axial post gadolinium T1, shows again the hydrocephalus. The diffuse attenuation of the periventriculated ependymal is well appreciated(arrow) Also posterior in the lateral ventricles there is a fluid-fluid level of debris (dashed arrow). Diffuse leptomeningeal attenuation is demonstrated. Imaging is in keeping with ventriculitis and meningitis.

**Pathology**


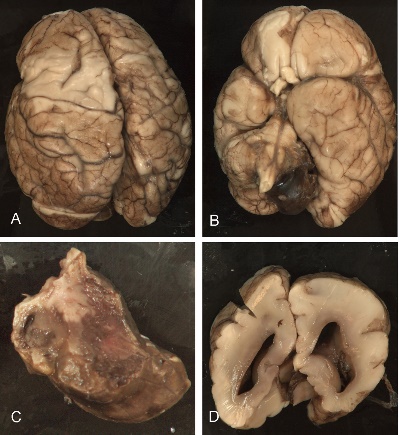

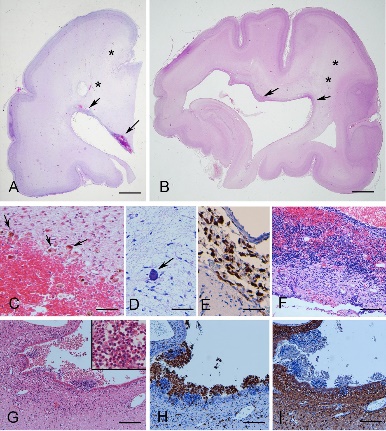


***Figure 3 &4***

Figure 3. Brain pathology A-B: macroscopically the brain showed a normal gyral pattern with some brown discolouration of the leptomeninges and a large cerebellar haemorrhage. C: hematoma in the left cerebellar hemisphere. D: coronal slice of the brain showing periventricular discolouration and intraventricular haemorrhages.

Figure 4. Brain pathology. A-B: low magnification photographs showing periventricular haemorrhages (arrows) with ventricular enlargement and normal cortical cytoarchitecture (asterisks indicate multiple areas of subcortical infarction). C, D: areas of and subcortical infarction with haemorrhages (arrows in C indicate hemosiderophages; arrow in D indicate a focus of mineralization). E: abundant meningeal macrophages (CD68 staining). F: haemorrhages within the germinal matrix. G-I: periventricular area with damage of ependymal cells, focal extrusion of macrophages (H: CD68 staining), glial scarring (I: glial fibrillary acidic protein staining) and intraventricular debris and focally evidence of ventriculitis with mixed population of macrophages and neutrophils (insert in G).
